# Association of football athlete engagement profiles with adolescent mental health — a latent profile analysis

**DOI:** 10.1186/s12889-025-23239-5

**Published:** 2025-06-02

**Authors:** Huarui Huang, Xiaoqi Sha, Chen Zhong, Ning Ma, Yizhou Shui

**Affiliations:** 1 School of Physical Education, Shaanxi Normal University, Xi’an, 710119 China; 2 School of Psychology, Shaanxi Normal University, Xi’an, China

**Keywords:** Latent profile analysis, Adolescent mental health, Team sports, Football

## Abstract

**Background:**

Adolescent mental health has become an important issue of global concern. Participation in sports, particularly team sports, is closely associated with improved adolescent mental health. However, the relationship between team sports participation and mental health is complex and varies across individuals. The current study was not able to clearly identify the mental health benefits associated with team sports among different individuals.

**Methods:**

A cross-sectional study was conducted across 64 schools in China, involving a total sample of 1,659 adolescents (M age = 12.51 years, SD = 2.285). Athlete engagement was assessed using the Athlete Engagement Questionnaire (AEQ), which measures four dimensions: self-confidence, vigor, dedication, and enthusiasm. Latent Profile Analysis (LPA) was employed to identify distinct athlete engagement profiles in football. In addition, the association between the identified latent profiles and major demographic characteristics was examined using the robust three-step method (R3STEP). Based on the Dual-Factor Model (DFM) of mental health, adolescents; mental health status was assessed using the Self-Esteem Scale and the Self-Rating Depression Scale (SDS). Finally, the relationship between different athlete engagement profiles and mental health outcomes was analyzed using the three-step Bolck-Croon-Hagenaars (BCH) method.

**Results:**

Latent Profile Analysis revealed that a three-profile model was most suitable: high engagement profile (Class 1, 46.2%, *n* = 766), moderate engagement profile (Class 2, 41.7%, *n* = 692), and low engagement profile (Class 3, 12.1%, *n* = 201). Furthermore, gender, level of school, and residence were key demographic predictors of profile membership. However, weekly training duration and years of football experience were not predictors of profile differences. Moreover, compared to the moderate and low engagement profiles, the high engagement profile exhibited the highest self-esteem and lowest depression symptoms.

**Conclusion:**

Application of LPA allows for a more differentiated understanding of how football participation impacts adolescent mental health within homogeneous subgroups. This, in turn, provides a theoretical basis for formulating personalized and multi-level intervention measures.

## Background

With the increasing social pressures and mental health issues, adolescent mental health has become a growing global concern [1]. Studies indicated that 10–20% of adolescents worldwide experience mental health problems [2, 3]. Adolescents’ mental health is of great importance for their daily study, social interaction and family functioning, and for developmental trajectories of mental health through adulthood [4, 5]. Ideally, early intervention would prevent the onset of mental health problems in adolescents [2]. However, the majority of adolescents lack the necessary knowledge and experience to smoothly overcome mental health problems [6]. Therefore, it is important to develop targeted measures and evidence-based interventions to help young people cope with stress and different challenges [7].

Mental health is defined by the World Health Organization (WHO) as a state of well-being in which the individual realizes his or her own abilities, can cope with the normal stresses of life, can work productively and fruitfully, and is able to make a contribution to his or her community, and is not merely the absence of mental illness [8]. Based on this definition, some studies have proposed the dual-factor model (DFM) of mental health [9–11]. The DFM postulates that mental health comprises two distinct but intertwined dimensions: psychopathology and well-being [9]. According to this model, researchers should concurrently examine both negative and positive indicators to address mental health in a comprehensive manner [12]. Under DFM, depression and self-esteem are considered effective negative and positive mental health indicators in the adolescent population in China [12]. They are also among the most commonly used negative and positive mental health indicators to evaluate the impact of sports on adolescent mental health [13].

Participation in sports is significantly associated with mental health outcomes [1, 14]. Compared with individual sports or other physical activities, participation in team sports is associated with better mental and social outcomes [15]. Specifically, compared to those participating in individual sports, adolescent athletes participating in team sports tend to report fewer depressive symptoms [16] and higher self-esteem [1]. Among the many team sports, football is the sport with the largest amount of participation globally [17] and is also one of the most popular team sports among adolescents. At the same time, there has been a growing interest in the use of football as a vehicle for mental health intervention [18]. Participation in football has been found to be positively associated with mental health and self-esteem [19].

Although studies on the relationship between various team sports (including football) and adolescent mental health have been increasing, the majority of studies still use variable-oriented approaches. There is a lack of person-oriented approaches in previous research on the relationship between sport participation in adolescent mental health. Variable-oriented research methods tend to emphasize average trends, which may overlook the potential differences among individuals [20]. Not all sports participants experience identical outcomes in the same way [21]. In terms of football participation, football training has varying effects on different individuals [22]. In contrast, this different experience highlights that relying solely on variable-oriented approaches may not adequately reveal the complex the relationship between team sports participation in adolescent mental health. A person-oriented approach treats individuals as the primary unit of analysis, rather than focusing solely on variables [21]. This approach allows for a more comprehensive understanding of distinct subgroups, rather than analyzing variables separately or focusing exclusively on mean-level trends [21].

When applying the person-oriented approach, Latent Profile Analysis (LPA) offers unique advantages [23]. LPA can identify differentiated effect characteristics from the features of the sample itself [24] and highlight hidden differences within groups, thereby recognizing distinct subgroups within the population [25]. As a result, LPA has been increasingly used by researchers in the field of sport psychology in recent years [21, 26, 27]. Despite the important contributions of these studies, the majority of them have had a relatively narrow focus, limited to models of self-determined motivation [28], have a relatively narrow focus, without distinguishing different profiles of sports participants. Furthermore, there has been a lack of study of the mental health differences among adolescents involved in different profiles of team sports participation. It is crucial to understand these differences as it can help in tailoring interventions and support on basis of the specific mental health needs of adolescents in different subgroups.

In China, athlete engagement is considered not only a useful means for objectively predicting the duration of sports participation as well as its attitude [29], but also an important indicator of an athlete’s positive mental aspects, reflecting their mentally healthy state [30]. Athlete engagement is defined as an enduring, positive cognitive and emotional experience in sports, with confidence, dedication, vigor, and enthusiasm as the main characteristics [31, 32]. To be exact, the dimensions are: confidence, defined as the belief in the capacity to reach a high level of performance and the desired goals; vigor, referring to the sensation of physical and mental vitality; dedication, reflected in the desire to invest time and effort into achieving goals that the individual considers important; and enthusiasm, related to strong emotions and high levels of enjoyment [31, 32]. Overall, athlete engagement is not limited to a specific form of participation (e.g., informal play, after-school activities, physical education classes, or organized club sports), but rather represents a positive experience and, as well as a positive attitude toward sport. It is appropriate to use athlete engagement to distinguish between different profiles of sports participants.

Therefore, this study intends to identify distinct athlete engagement profiles among adolescent football participants using LPA, based on the four dimensions of engagement. Furthermore, this study seeks to fill a research gap by examining the relationship between team sports and adolescent mental health from a person-centered perspective. Although there is a general consensus that participation in team sports is associated with improved social and mental health, independent of the type of team sport, age of participants [33], it remains essential to investigate the demographic predictors of different engagement profiles. Such predictors can serve as meaningful indicators for identifying individuals who may benefit from targeted interventions. Specifically, our first aim is to identify through LPA different profiles of athlete engagement in football among adolescents. The second aim is to examine which demographic variables are associated with these latent profiles. As a third aim, we will examine how these different profiles of athlete engagement in football are related to key mental health indicators, namely depression and self-esteem, within the framework of the DFM.

## Methods

### Participants and procedure

This cross-sectional study adopts the convenience sampling method, with 1,692 participants recruited from 64 schools across five provinces in China. The selected participants were adolescents aged 8–16 (M age = 12.51 years, SD = 2.285) who had more than one year of football training experience and engaged in at least one hour of weekly training duration. However, they were not professional adolescent football players, but rather engaged in football training during their leisure time. Students who did not play football, as well as individuals with physical disabilities, diagnosed mental health conditions, or sports-related injuries, were excluded from the study.

The study protocol was approved by the Ethics Committee of Shaanxi Normal University (No. 202516002). In this study, we Informed consent was obtained from all the participants and their legal guardians. With permission from each school, questionnaires were administered to classes of adolescents. The principles of voluntariness, confidentiality, and non-harmfulness were strictly adhered to throughout the study. Prior to participation, the purpose, content, and precautions of the survey were fully explained to the participants, and written informed consent was obtained. During the administration of the questionnaires, two researchers were always present to provide information on how to fill out the questionnaires and to resolve any doubts that might arise during the process. Participants were allowed to withdraw from the survey at any time during the process. Each questionnaire was coded and entered anonymously, and all data and information were kept confidential and were not disclosed to any external parties, except for the purpose of scientific research.

## Measurement

### General information

The general information questionnaire collected demographic data, including gender (1 = male, 2 = female), place of residence (1 = urban, 2 = rural), level of school (1 = primary school, 2 = junior high school, 3 = senior high school), weekly football training duration, and years of football experience.

### Athlete engagement questionnaire (AEQ)

The Questionnaire, compiled by Lonsdale and Jackson [31], was used to gather data. The AEQ was revised and translated into Chinese by Zhang [34]. It has been shown to have good reliability and validity among Chinese primary and secondary school students [35]. The AEQ includes 16 items in four dimensions: self-confidence (Items 1–4), vigor (Items 5–8), dedication (Items 9–12), and enthusiasm (Items 13–16). To ensure the relevance of the measurements, the original questionnaire limited the scope of comparison to football. The questionnaire used a 5-point Likert scale, ranging from 1 (never) to 5 (always). In this study, Cronbach’s α of the AEQ was 0.95. The Cronbach’s α of four dimensions—self-confidence, vigor, dedication, and enthusiasm—was 0.88, 0.85, 0.87, and 0.88.

### Self-esteem scale

The Self-Esteem Scale was used to evaluate participants’ level of self-esteem [36]. Translated into Chinese by Wang et al. [37], and its validity and reliability have been confirmed in children and adolescents aged 4 to 18 years [38]. The scale consists of 10 items, with each item rated from 1 (not very true of me) to 4 (very true of me). Among them, 3, 5, 8, 9, 10 are positive scores, and the other 5 entries are reverse scores. Cronbach’s α coefficient was 0.82 at the time of this survey.

### Self-rating depression scale (SDS)

The scale, compiled by Zung [39], was used to assess depressive symptoms. The Chinese version has been validated among students from Grade 2 in primary school to Grade 11 in high school (ages 8–16), showing good psychometric properties [40]. It consists of 20 items, rated on a 4-point scale from 1 (no or little time) to 4 (most or all of the time). A total SDS score was calculated by first reversely scoring items of 2, 5, 6, 11, 12, 14, 16, 17, 18, and 20, The Cronbach’s α coefficient of SDS was 0.79.

### Statistical analyses

This study employed SPSS 28.0 and Mplus 8.3 software for data management and analysis. Initially, descriptive statistical analysis and Pearson correlation analysis were conducted to examine the study variables. We then used LPA to identify the athlete engagement profiles of adolescents participating in football. We compared the fit indices of the models with two to four profiles to determine the number of profiles that best fit the data. Based on the standardized processing of the data, models with one to four profiles were estimated using the robust maximum likelihood estimator available in Mplus 8. To avoid potential issues from converging on suboptimal local maxima, 5,000 random sets of start values were estimated with 1,000 iterations, and the 200 best solutions were retained for final optimization [41]. We used indicators such as the Akaike Information Criterion (AIC), Bayesian Information Criterion (BIC) [42], sample size Adjusted Bayesian Information Criterion (ABIC) [43], Likelihood Ratio Test (LRT), Bootstrap Likelihood Ratio Test (BLRT) [44], and Entropy for model comparison [45]. Models with lower values of AIC, BIC, and ABIC were considered to have better fit, and among competing models, the model exhibiting the lowest values across these criteria was selected as optimal. The significant LRT and BLRT values suggest that the fit of the k-profile model is significantly better than that of the k-1 profile model. Entropy highlights the precision of the classification with values ranging from 0 (lower accuracy) to 1 (higher accuracy), with values greater than 0.8 indicating a classification accuracy above 90% [46]. In addition, the smallest proportion of profile cut-off was 0.05 [47].

In addition, a latent multinomial logistic model that regressed the latent profiles variable on major demographic characteristic was estimated using the robust three-step method (R3STEP) [48, 49]. Finally, to examine how the profiles are associated with adolescent mental health, the three-step Bolck-Croon-Hagenaars (BCH) method was used [50]. First, the latent profiles were estimated. Then, respondents were assigned to their most likely profile. And finally, differences between these profiles in distal outcomes were estimated. At the same time, we corrected for the nested structure of the data by estimating cluster-robust standard errors.

## Results

### Basic characteristics

A total of 1,692 adolescents from primary school to senior high school participated in the study. Finally, 1,659 participants completed the full survey without missing responses, yielding a validity rate of 98.0%. The detailed characteristics of participants are shown in Table 1.

**Table 1:** Demographic information of participants

Variables	Frequency	Percentage
Gender		
Male	1135	68.4%
Female	524	31.6%
level of school		
Primary school students	885	53.1%
Junior high school students	543	32.6%
Senior high school students	231	14.3%
Place of residence		
Urban areas	905	54.6%
Rural areas	754	45.4%
Weekly training duration		
1 ~ 8 h	1047	63.1%
9 ~ 28 h	612	36.9%
Football experience		
1 ~ 5 years	1265	76.3%
6 ~ 12 years	394	23.7%

### Common method deviation test

To examine common method bias, this study applied Harman’s single-factor test [51]. The exploratory factor analysis revealed nine factors with eigenvalues greater than 1. The first factor explained 27.1% of the variance, which is below the critical threshold of 40%, indicating that common method bias is not a serious concern in this study [52].

### Descriptive statistics and correlation analysis

Descriptive statistics and correlation analysis for each variable are presented in Table 2. The results of the correlation analysis indicate that all variables, except for depression, are significantly positively correlated with each other, while depression shows a significant negative correlation with all other variables (all *p* < 0.001).

**Table 2:** Descriptive statistics and correlation analysis (*N* = 1659)

	1	2	3	4	5	6
**Athlete Engagement**						
1. Self-Confidence	1					
2. Vigor	0.68***	1				
3. Dedication	0.74***	0.76***	1			
4. Enthusiasm	0.63***	0.75***	0.74***	1		
**Mental Health**						
5. Self-Esteem	0.55***	0.48***	0.49***	0.48***	1	
6. Depression	-0.36***	-0.38***	-0.37***	-0.39***	-0.57***	1
M	4.00	4.19	4.25	4.36	3.16	2.06
SD	0.80	0.73	0.72	0.71	0.51	0.36

Note: The Mean (M) and Standard Deviation (SD). *, *p* < 0.05; **, *p* < 0.01; ***, *p <* 0.001

### Model fitting

We analyzed the number of profiles in the sample based on the four dimensions of athlete engagement. The fit indices for each model are presented in Table 3. The AIC, BIC, and ABIC values all decreased progressively from the two-profile solution to the four-profile solution. However, in the four-profile, one Class contained only 1.3% of the participants (less than 5%), and the LRT value for the 4-profile was not significant, therefore it was excluded. The Entropy values for the two-profile and three-profile models were both greater than 0.08, with the three-profile model having a higher Entropy value of 0.90, indicating high classification accuracy, particularly for the three-profile model. The LRT and BLRT values from the two-profile solution to the three-profile solution were significant, showing that each additional profile significantly improved the model fit compared to the previous one. Overall, the three-profile solution showed better model fit than the two-profile model. This was evidenced by lower AIC and BIC values, higher entropy, and significant LRT and BLRT results. Therefore, the three-profile model is considered the optimal model for this study. Furthermore, the three-profile solution had very high average latent class probabilities (> 0.94) for the most likely latent class membership (see Table 4), indicating that with the three-profile solution, profile membership can be estimated with a high degree of accuracy.

**Table 3:** Fit indices for LPA

Model	AIC	BIC	ABIC	Entropy	LRT	BLRT	The proportion of each profile
1 profile	18844.15	18887.46	18862.05				100%
2 profiles	15886.24	15956.62	15915.32	0.85	0	0	43.1%,56.9%
**3 profiles**	**14364.85**	**14462.30**	**14405.12**	**0.90**	**0**	**0**	**46.2%**,**41.7%12.1%**
4 profiles	14041.75	14166.28	14093.21	0.91	0.23	0	45.3%,40.2% 13.%,1.3%

**Table 4:** Latent class probabilities for most likely membership

	Class 1	Class 2	Class 3
1	**0.96**	0.04	0
2	0.04	**0.94**	0.02
3	0	0.05	**0.95**

### Class assignments

**Fig. 1:**
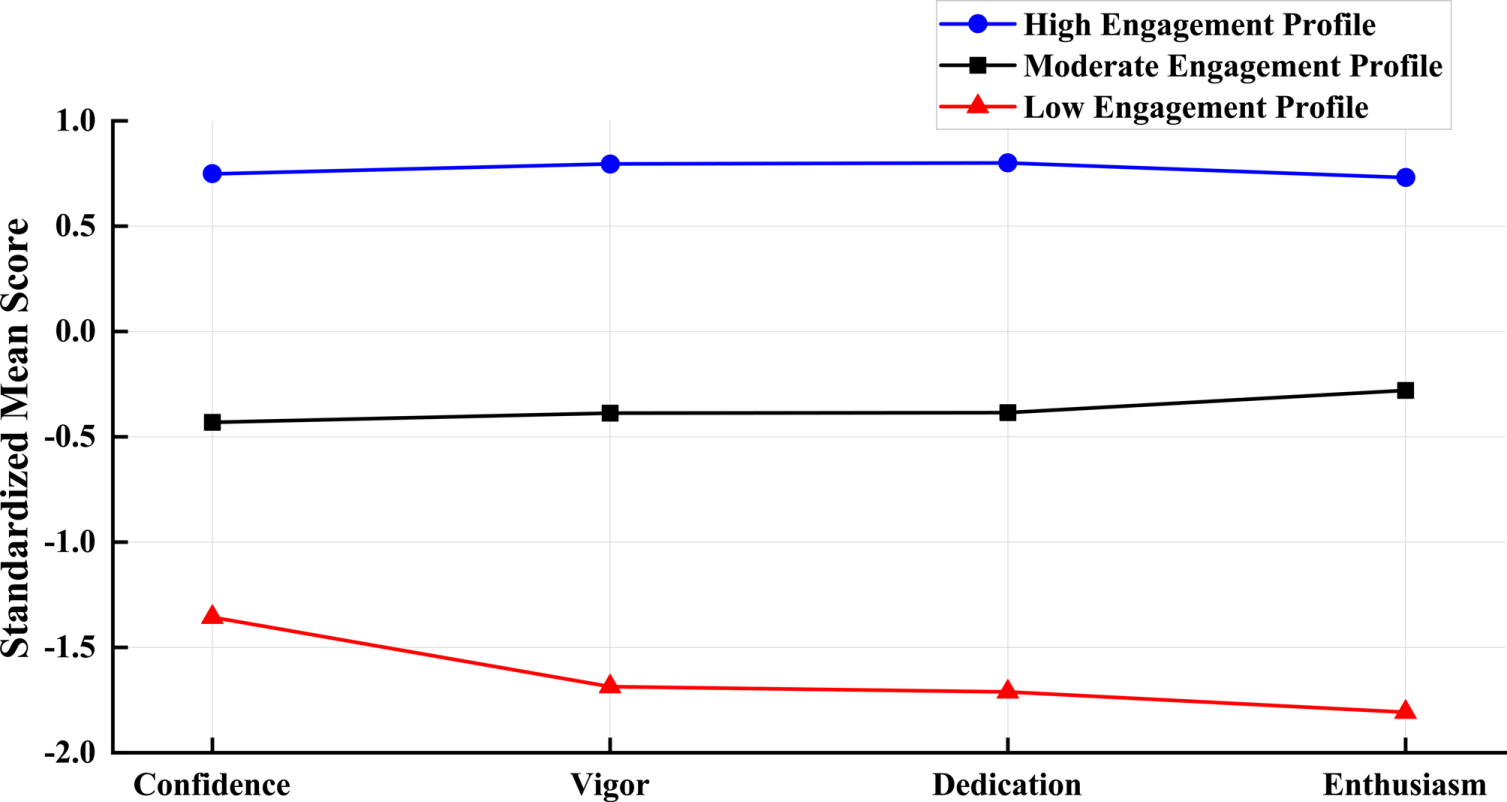
Mean scores in the three latent profiles (standardized scores)

Figure 1 depicts the estimated averages for the three profiles across the four dimensions of athlete engagement. Class 1 (46.2%, *n* = 766) exhibited the highest scores across all dimensions of athlete engagement and was therefore named *the high engagement profile*. Class 2 (41.7%, *n* = 692) demonstrated relatively balanced scores across various dimensions of athlete engagement, all of which were maintained at moderate levels. Therefore, it was named *the moderate engagement profile*, Class 3 (12.1%, *n* = 201) had the lowest scores across various dimensions of athlete engagement and was therefore named *the low engagement profile*.

### Multinomial logistics

Multinomial logistic regression using the R3STEP was conducted to analyze the relationship between demographic characteristics and the latent profiles of athlete engagement. The dependent variable was the latent profiles of athlete engagement, while the independent variables included gender, residence, level of school, weekly football training duration, and years of football experience. The low engagement profile was used as the reference profile. The results are summarized in Table 5. Compared to adolescents in the low engagement profile, males were more likely to be in the high engagement profile (OR = 0.55, 95% CI [0.46, 0.65], *p* < 0.001) and the moderate engagement profile (OR = 0.72, 95% CI [0.60, 0.86], *p* < 0.001). Similarly, adolescents from urban areas were more likely to be in the high engagement profile (OR = 0.71, 95% CI [0.56, 0.89], *p* = 0.001), and the moderate engagement profile (OR = 0.80, 95% CI [0.63, 1.02], *p* = 0.047), compared to those in the low engagement profile. While level of school did not significantly differentiate between the moderate and low engagement profiles (OR = 1.03, 95% CI [0.84, 1.25], *p* = 0.800), it was a significant predictor when comparing the high engagement profile to the low engagement profile (OR = 0.70, 95% CI [0.58, 0.84], *p* < 0.001). In contrast, weekly training duration and years of football experience were not significantly associated with different profiles in either comparison. For training hours per week (C2 vs. C3, *p* = 0.790; C1 vs. C3, *p* = 0.969). For training years (C2 vs. C3, *p* = 0.969; C1 vs. C3, *p* = 0.143).

**Table 5:** The results of multinomial logistics regression

	Class 2 (*n* = 692)		Class 3 (*n* = 201)	
	OR	95% CI	OR	95% CI
gender	0.72***	0.60, 0.86	0.55***	0.46, 0.65
residence	0.80*	0.63, 1.02	0.71**	0.56, 0.89
level of school	1.03	0.84, 1.25	0.70***	0.57, 0.86
Training per week	1.03	0.83, 1.28	1.14	0.92, 1.42
Football experience	1.00	0.77, 1.29	1.23	0.96, 1.58

Note: Reference group is Class 1 (*n* = 766). Odds ratio (OR). 95% confidence interval (95% CI). *, *p* < 0.05; **, *p* < 0.01; ***, *p <* 0.001 (standardized scores)

### Association between profiles of athlete engagement and adolescent mental health

The results are presented in Tables 6 and 7. Table 6 reports the standardized mean scores and standard errors (SE) of self-esteem and depression for each of the three athlete engagement profiles. Adolescents in the high engagement profile reported the highest level of self-esteem (M = 0.56, SE = 0.03) and the lowest level of depression (M = − 0.47, SE = 0.04). In contrast, adolescents in the low engagement profile demonstrated the lowest level of self-esteem (M = − 0.94, SE = 0.05) and the highest level of depression (M = 0.84, SE = 0.05). The moderate engagement profile showed intermediate scores for both self-esteem (M = − 0.35, SE = 0.03) and depression (M = 0.27, SE = 0.03).

**Table 6:** Mental health outcomes per athlete engagement profile

	Class 1		Class 2		Class 3	
	M	SE	M	SE	M	SE
Self-Esteem	0.56	0.03	-0.35	0.03	-0.94	0.05
Depression	-0.47	0.04	0.27	0.03	0.84	0.05

Note: The Mean (M) and Standard Error (SE) (standardized scores)

Table 7 presents the results of pairwise comparisons between profiles, including chi-square statistics (X^2^), estimate, effect sizes (Cohen’s d), and corresponding 95% confidence intervals (CI). For self-esteem, adolescents in the high engagement profile reported significantly higher scores compared to those in the moderate (X^2^ = 338.09, Estimate = 0.91, p < 0.001, Cohen’s d = 1.00, 95% CI [0.90, 1.11]) and low engagement profiles (X^2^ = 555.34, Estimate = 1.51, p < 0.001, Cohen’s d = 1.66, 95% CI [1.49, 1.83]). Similarly, adolescents in the moderate engagement profile showed significantly higher self-esteem compared to those in the low engagement profile (X^2^ = 83.73, Estimate = 0.60, p < 0.001, Cohen’s d = 0.70, 95% CI [0.54, 0.86]). For depression, adolescents in the high engagement profile reported significantly lower scores compared to those in the moderate (X^2^ = 201.94, Estimate = − 0.74, p < 0.001, Cohen’ s d = − 0.76, 95% CI [–0.87, − 0.66]) and low engagement profiles (X^2^ = 406.89, Estimate = − 1.31, *p* < 0.001, Cohen’s d = − 1.31, 95% CI [–1.48, − 1.15]). Additionally, adolescents in the moderate engagement profile reported significantly lower depression scores compared to those in the low engagement profile (X^2^ = 78.25, Estimate = − 0.57, *p* < 0.001, Cohen’s d = − 0.64, 95% CI [–0.79, − 0.49]).

**Table 7:** Differences in mental health across athlete engagement profiles

		X^2^	Estimate	Cohen’s d	95% CI
**Self-Esteem**	C 1 VS C2	338.09	0.91	1.00***	0.90, 1.11
	C 1 VS C3	555.34	1.51	1.66***	1.49, 1.83
	C 2 VS C3	83.73	0.60	0.70***	0.54, 0.86
**Depression**	C 1 VS C2	201.94	–0.74	–0.76***	–0.87, − 0.66
	C 1 VS C3	406.89	–1.31	–1.31***	–1.48, − 1.15
	C 2 VS C3	78.25	–0.57	–0.64***	–0.79, − 0.49

Note: Chi-square test statistic (X^2^). The Effect Sizes (Cohen’s d). 95% confidence interval (95% CI). *, *p* < 0.05; **, *p* < 0.01; ***, *p <* 0.001 (standardized scores)

## Discussion

The first aim of this person-oriented study was to identify latent profiles of football athlete engagement. According to the results, three profiles were identified: high engagement, moderate engagement, and low engagement profile. They are characterized by different degrees of sports experience and different attitudes toward sports, specifically reflected in four aspects: self-confidence, dedication, vigor, and enthusiasm.

The majority of adolescents participating in football were in the high and moderate engagement profiles, with slightly more in the high engagement profile. This suggests that most adolescents have a positive attitude toward football and a positive sports experience. Enjoyment is an important factor motivating adolescents to actively participate in sports [53]. Football has high levels of enjoyment for children and adolescents [54–56]. Furthermore, the role of physical education in fostering a passion for sports is also pivotal [57]. The Chinese government has implemented measures to promote widespread participation in football, which include integrating football into school curricula and supporting youth football through competitions and training programs [58].

The small sample size of the low engagement profile suggests that only a minority of adolescents lack a positive attitude toward football and have a negative sports experience. The reasons for this outcome seem to be explainable from several aspects. In China, although campus football has been strongly promoted by the central government, there is still a serious shortage of professional football physical education teachers, and since campus football courses are typically conducted in large classes, it becomes difficult for teachers to pay attention to all students [59]. Moreover, since 2017, football has become one of the selective sports for physical fitness tests in many regions of China, and in numerous schools, football performance is also used as a an indicator to evaluate students’ annual achievement [60]. This policy leads some students to participate in football activities primarily because they need to meet the physical fitness test standards and fulfill the annual evaluation requirements, rather than their genuine interest in football, thus resulting in a lack of intrinsic positive mental motivation.

In addition, we found minimal differences in the self-confidence dimension between the low, moderate, and high engagement profiles, while more significant differences were observed in the other dimensions. Previous study indicated that under the football education model in China, even students with lower football skills can improve their self-confidence and self-esteem, ensuring that they are not excluded from football due to differences in athletic ability [61]. Furthermore, self-confidence in team sports is often influenced by the actions of teammates [62]. In this study, the positive actions of students in the moderate and high engagement profiles may have positively impacted the self-confidence of those in the low engagement profiles. This could explain why the gap in self-confidence between the low, moderate, and high engagement profiles was relatively small.

The second aim of this study was to identify the demographic variables associated with the latent profiles of athlete engagement. The results indicated that gender, level of school, and residence were key demographic predictors of profile membership. However, weekly training duration and years of football experience were not predictors of profile differences.

We found that, compared to adolescents in the low engagement profile, males were more likely to be in the high engagement profile and the moderate engagement profile. Previous study has shown gender differences in football participation among adolescents [58]. Specifically, males generally exhibit more positive attitudes toward sports, participate more frequently, and maintain higher levels of physical activity compared to females [63]. Socially, there is a widespread preference for men’s football, which is often perceived as more competitive and entertaining [58]. On a personal level, concerns related to body image and identity have been associated with girls’ lower participation in sports that are perceived as aggressive or masculine in nature [64].

Similarly, adolescents from urban areas were more likely to be in the high or moderate engagement profiles compared to those in the low engagement profile. Previous studies have shown that sports facilities around the home, accessibility to sports venues, and the type and number of sports facilities that are suitable for children and adolescents influence children and adolescents’ participation in sports programs [65, 66]. In China, significant urban-rural disparities exist in the provision of sports infrastructure [67]. In addition, under the dualistic urban-rural structure unique to China, urban residents tend to possess higher socioeconomic status than their rural counterparts [68]. Parents with higher socioeconomic status are generally more supportive of their children’s participation in sports, as reflected in greater parental involvement and the provision of necessary resources [69, 70].

In addition, as the level of school increases, adolescents are more likely to be classified into the low engagement profile. This trend is primarily driven by changes in academic demands from primary to secondary education. On the one hand, football training in primary school is mainly oriented toward interest cultivation and general physical development. In contrast, at the secondary level, football has become part of the physical fitness tests and is also used as an indicator for evaluating students’ annual academic performance [60]. On the other hand, compared to secondary school students, primary school students typically face lower academic pressure. This relatively lighter academic burden creates favorable conditions for sustained participation in physical activities [58]. In contrast, the increased academic demands in secondary school reduced time and fewer opportunities for physical activities, adversely affecting their involvement in sports [71, 72].

The third aim of this study was to investigate the association between different profiles of football athlete engagement and mental health indicators, namely self-esteem and depression, within the framework of the DFM. The results showed that adolescents in the high engagement profile had significantly higher levels of self-esteem, as well as lower levels of depression, compared to the moderate and low engagement profiles.

The DFM provides a perspective to interpret differences in mental health outcomes across different engagement profiles. Previous study has demonstrated that, within the DFM framework, the mental health status of Chinese adolescents can be categorized into three heterogeneous groups (Flourishing, Vulnerable, and Troubled) [12]. This contrasts with the four discrete mental health groups typically identified in Western contexts (Flourishing, Troubled, Vulnerable, and Symptomatic but content) [73]. Adolescents in the high engagement profile are expected to exhibit a mental health characteristics characterized by high self-esteem and low levels of depression. In the DFM framework, this profile corresponds to the Flourishing group, reflecting optimal mental health [12]. Those in the moderate engagement profile demonstrate intermediate mental health outcomes. Within the DFM framework, their characteristics is similar to the Vulnerable group. This suggests that their positive mental health indicators are average. Adolescents with a low engagement profile exhibit characteristics similar to the Troubled group in the DFM framework, reflecting lower levels of mental health [12]. These findings may be explained from several aspects.

Adolescents in the high engagement, compared with those in the moderate and low engagement profiles, have a more fulfilling and positive sports experience and a more positive attitude toward sports. Previous studies have shown that a positive attitude toward sports is associated with higher levels of self-esteem and self-confidence, lower levels of anxiety and depression, and better overall mental health status, whereas a negative attitude is associated with reduced participation and poorer mental health outcomes [74]. Additionally, among adolescent football participants, higher levels of athlete engagement are associated with stronger team cohesion and more positive team relationships [75]. In collectivistic-oriented Asian contexts, adolescents may benefit more from good relationships [76]. These benefits include positive mental health outcomes within the framework of the DFM, such as higher self-esteem and lower levels of depression [12].

The observed differences in mental health among adolescents participating in football also be related to the inherent characteristics of football as a team sport. The mental health benefits of team sports, including football, also be associated with positive sports experiences. These benefits are inclusive of reducing social anxiety and enhancing self-esteem [77]. Furthermore, the cooperative nature of team sports provides adolescents with opportunities for peer interaction [78], which has been negatively associated with depressive symptoms and positively associated with self-esteem [1]. The differences in mental health levels observed among adolescents participating in football are also closely related to adolescent developmental stage. During the period when adolescents have to be confronted with the dual challenges of academic pressure and peer relationship stress. Within the framework of DFM, increasing levels of academic and interpersonal stress are recognized as risk factors associated with negative mental health status [12]. Participation in sports activities has been reported to be related to lower levels of perceived stress [1, 79, 80].

Moreover, athlete engagement, as a positive and meaningful experience, has been associated with sustained sports participation over time [81]. From a neuroscience perspective, long-term physical exercise has been linked to neurogenesis, angiogenesis, and synaptogenesis, which may be related to lower levels of depression and anxiety. In addition, the central nervous system, accountable for neuromuscular activities and task execution, offer anticipatory control that could help ward off mental depression and anxiety [82]. Exercise has also been associated with elevated endorphin secretion, which in turn has been linked to better mood and higher levels of self-esteem [1].

Transferring the findings into practical implications, with consideration given to inter-individual differences, helps to tailor interventions for specific subgroups [25, 28]. For example, for adolescents in the high engagement profile, it is important to maintain their positive engagement while being cautious of the pressure caused by overly high expectations, in order to avoid negative mental impacts [83]. Additionally, given the physiological characteristics of adolescents and the likelihood of player-to-player collisions in football, special attention should be paid to preventing the risks of sports injuries due to excessive exercise, as well as the physical and mental problems caused by sports injuries [84]. For adolescents in the moderate engagement profile, physical education teachers can set tiered goals based on individual levels, allowing them to experience a sense of achievement through moderate challenges, thereby fostering a more positive sports experience. Furthermore, during the teaching process, physical education teachers should enrich the content and create situations to further stimulate students’ motivation [85]. For adolescents in the low engagement profile, society, education and families should introduce the physical and social benefits of football to help shape a positive perception of the sport, thereby influencing adolescents’ willingness to participate in football activities [58]. At the same time, it is necessary to stimulate the interest of this profile of adolescents in football. Some schools have already provided successful examples by hosting football cultural events, and top-level celebrity footballers are invited to schools [60]. In summary, the results reveal the differences in mental health among adolescents involved in football at different athlete engagement profiles, providing new evidence for a deeper understanding of the multidimensional impact of team sports on adolescent mental health. Simultaneously, we also offer theoretical support for personalized and targeted interventions in team sports to improve adolescent mental health. Football can have a positive role to play in mental health promotion and in the delivery of interventions that positively impact mental health, but it should not be seen as a “wonder drug” that can be expected to work universally and under all circumstances [86]. Therefore, in the future, physical education teachers should develop personalized and targeted football intervention programs according to different types of adolescents to effectively promote adolescent mental health.

Despite all the relevant findings, several limitations of the current study should be noted. Firstly, as an initial attempt to investigate the impact of team sports on adolescent mental health based on the LPA, this study utilized a large sample covering adolescents across all age groups. Although there is a general consensus and consistent evidence that participation in team sports is associated with improved social and psychological health, independent of the type of team sport, age of participants, or somatic or mental health problems [33], the results of our study indicated that gender, level of school, and residence were key demographic predictors of profile membership. Therefore, future studies could focus on specific age ranges, such as primary school, middle school, or high school stages, so as to offer more precise evidence and support the development of personalized intervention strategies tailored to adolescents at different developmental stages.

Secondly, our study did not include factors related to coaches. Previous studies have highlighted the critical role of coaches in shaping adolescents’ sport experiences and influencing athlete engagement [87, 88]. However, no data concerning coaches, such as their behavior, qualifications, or the relationship between coaches and athletes, were collected in this study. This limitation restricted our ability to examine how coaching factors may interact with different athlete engagement profiles or mental health outcomes. Future research should include data from multiple sources, including variables that reflect the role and influence of coaches, to provide a more comprehensive understanding of how interpersonal and structural factors influence athlete engagement among adolescents.

Finally, this study adopted a cross-sectional design, which limits the interpretation of causal statements and prevents the investigation of temporal stability in the patterns. Although there is no direct evidence to suggest that adolescents’ athlete engagement profiles in sports change over time, current studies have shown that adolescents’ learning engagement undergoes dynamic changes over time [89]. Therefore, future study could focus on the dynamic evolution of athlete engagement profiles and further explore how changes in these profiles influence adolescents’ mental health, providing a more comprehensive understanding of the long-term effects and mechanisms of participation in team sports.

## Conclusion

In conclusion, the results of this study through LPA identified three subgroups of adolescent athlete engagement and further revealed differences in depression and self-esteem among these subgroups. Furthermore, gender, level of school, and place of residence were identified as key demographic predictors of profile membership. The analysis indicates that football, as a representative team sport, can promote adolescent mental health, but its effects vary across subgroups. This new approach helps generate target group-specific knowledge, which provides the basis for planning tailored multilevel programs to promote mental health more effectively through football among different adolescents.

## Data Availability

The data that support the findings of this study are available from the corresponding author upon reasonable request.

## Abbreviations

LPA: Latent profile analysis
DMF: Dual-factor model
AEQ: Athlete engagement questionnaire
SDS: Self-esteem scale and the self-rating depression scale
AIC: Akaike information criterion
BIC: Bayesian information criterion
ABIC: Sample size adjusted bayesian information criterion
LRT: Likelihood ratio test
BLRT: Bootstrap likelihood ratio test
BCH: The three-step bolck-croon-hagenaars
R3STEP: Three-step method
OR: Odds ratio
95% CI: 95% confidence interval
SE: Standard error
X^2^: Chi-square test statistic
Cohen’s d: The effect sizes
